# MRI Findings of Acute on Chronic Osteomyelitis of Tibia in a 12-Year-Old Child

**DOI:** 10.7759/cureus.67679

**Published:** 2024-08-24

**Authors:** Iram Saifi, Pallavi Kar, Shivali V Kashikar, Pratapsingh Parihar, Azeem I Saifi, Khizer Ansari

**Affiliations:** 1 Radiodiagnosis, Jawaharlal Nehru Medical College, Wardha, IND; 2 Medicine, Jawaharlal Nehru Medical College, Wardha, IND

**Keywords:** involucrum, sequestrum, trabecular bone loss, garre sclerosing osteomyelitis, staphylococcus aureus

## Abstract

Pediatric patients with osteomyelitis, a serious bone infection, have several difficulties. A 12-year-old child with an acute osteomyelitis diagnosis is the subject of this case study. The child had decreased limb function, a fever, and localized pain. Laboratory testing and diagnostic imaging procedures verified that Staphylococcus aureus was the culprit for the infection. Surgical debridement and intravenous antibiotics were used in combination for treatment. Therapy responses were constantly examined, and modifications were made in response to clinical and radiological findings. Prompt intensive treatment and early detection were essential for controlling the infection and averting long-term consequences. This example emphasizes the value of a multidisciplinary approach to treating pediatric osteomyelitis, pointing out possible directions for future study and presenting best practices.

## Introduction

The skeleton is the framework of the human body. It is divided into the axial and appendicular skeleton, which helps with movement. Any infection affecting the bones can physically, socially, and financially impede human growth. If an infection occurs in one of the 206 bones of the human body, it can affect not only the movement of the body but also increase the burden of disability in society. Osteomyelitis is one of the leading infections affecting bones in the Indian population, predominantly in the lower extremities of young children. It is the most common infection in the rural population [1].

## Case presentation

A 12-year-old child presented with the complaint of an ulcer on the right leg on the shin of the tibia. As narrated by the father of the patient, the child was asymptomatic four years ago when he developed a boil on his right leg, which gradually burst and ulcerated till the underlying bone was visible on the floor of the ulcer. The child was taken to a local hospital and dressing was done. One year back, the child had a similar complaint of a boil on the right lower leg for which dressing was done. The child had repeated bouts of fever in between the recurrent episodes. In the present scenario, the patient also complained of fever and pus discharge from the ulcerated site for 10 days. It was associated with pain for which he was unable to walk for two months.

On general examination, pulse rate was 92 beats per minute and blood pressure was 106/80 mmHg. There was no cyanosis, pallor, icterus, or lymphadenopathy. On local examination, an ulcer was present on the right shin of the tibia. The surrounding margins of the ulcer were edematous and inflamed. The underlying bone was visible on the base of the ulcer. There was associated pus discharge, which was yellow, for which no biopsy was done. There was slight local tenderness. The cardiovascular, nervous, respiratory, and gastrointestinal systems were within normal limits. The patient was advised X-ray but the findings were not significant so the patient was advised MRI of the right lower limb. 

**Figure 1:**
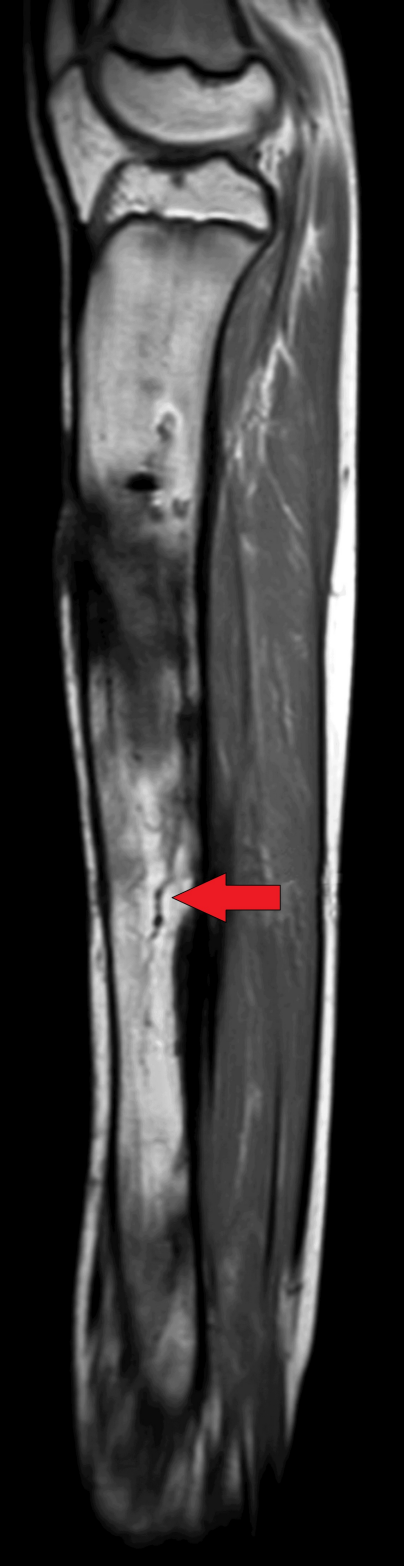
Sagittal T1WI MRI of tibia showing linear hypointense irregularities in the tibial diaphysis suggestive of areas of cortical erosion T1W1: T1-weighted imaging

**Figure 2:**
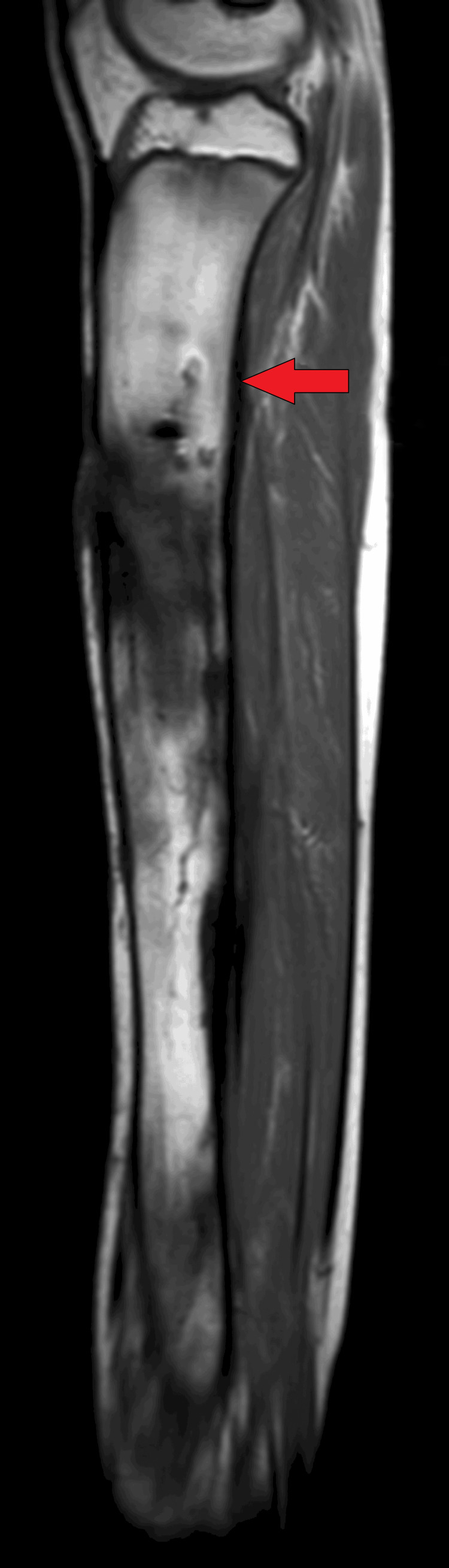
Sagittal T1WI Right tibia white arrow showing hypointense thickened periosteum T1W1- T1 weighted imaging

**Figure 3:**
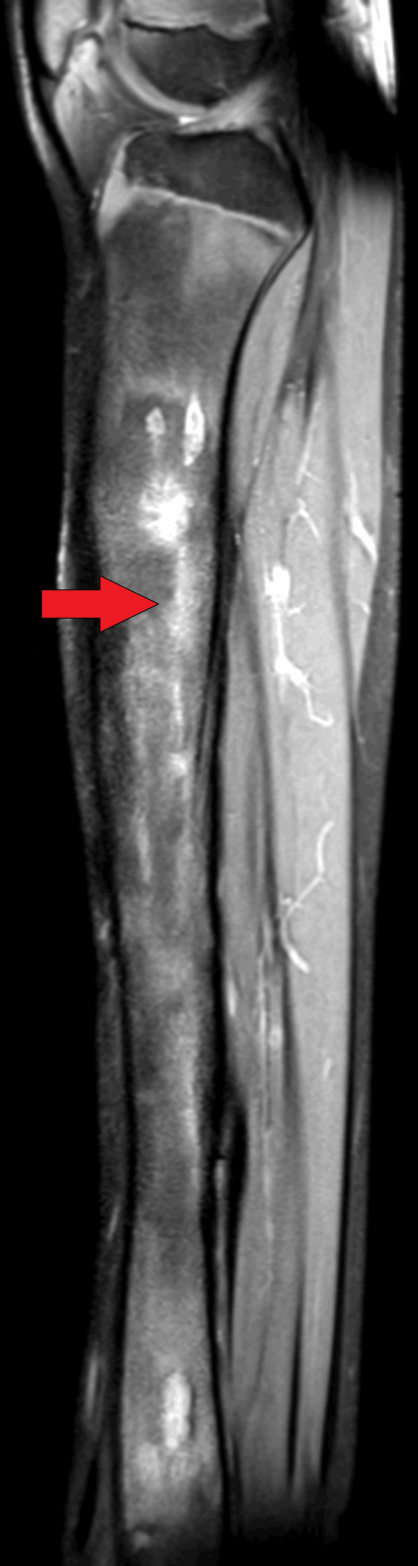
Sagittal PD-Fat-Sat MRI showing extensive hyperintensity involving the marrow of the diaphysis of tibia suggestive of marrow edema PD-Fat-Sat: proton density fat suppression image

**Figure 4:**
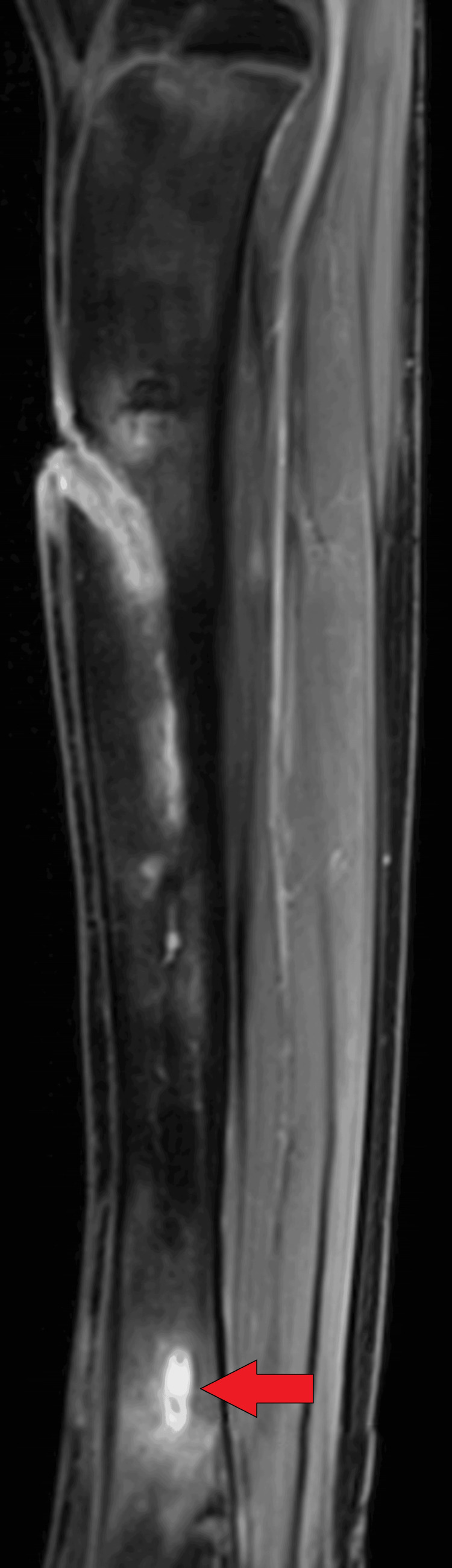
Sagittal T1WI contrast image showing tiny focal areas of post-contrast enhancement involving the lower tibial diaphysis suggestive of intraosseous abscess T1W1: T1-weighted contrast imaging

**Figure 5:**
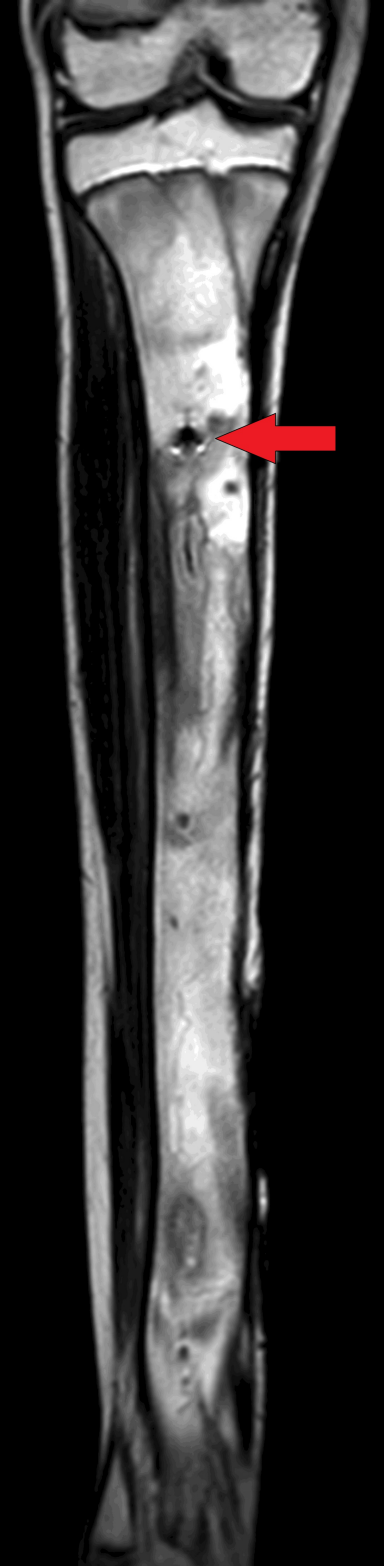
Coronal T1WI MRI of the tibia showing small areas of T1WI hypointensity, which do not show any enhancement in marrow suggestive of sequestrum. T1W1: T1-weighted imaging

On MRI T1WI sagittal image, there was evidence of cortical irregularity with areas of cortical destruction appearing hypointense on T1WI and involving the diaphysis of the tibia suggestive of cortical erosions (Figure 1). There was associated periosteal thickening noted, suggestive of periosteal reaction (Figure 2). There was a thick-walled sinus tract noted involving the diaphysis of the tibia reaching up to the skin surface in the proximal-mid third of the tibia appearing hyper-intense on T2-weighted imaging/proton density fat suppression(T2WI/PDFS), hypointense on T1WI and showing thick post-contrast enhancement of the sinus tract wall suggestive of active sinus tract. There was extensive T2WI/PDFS (proton density fat suppression image) hyperintensity involving the marrow of the diaphysis of the tibia suggestive of marrow edema (Figure 3). A tiny focal area of post-contrast enhancement was noted involving the tibial diaphysis, which appeared hyper-intense on T2WI/PDFS images suggestive of an intraosseous abscess (Figure 4). Small areas of T1WI and T2WI hypo-intensities did not show enhancement in marrow suggestive of sequestrum (Figure 5). In the lower one-third of the tibia, there were small intraosseous abscesses that showed cloaca formation opening into the adjoining muscle plain posteriorly appearing as small gaps in the cortex of bone.

The patient was diagnosed with a case of acute chronic osteomyelitis with intraosseous abscesses and sinus tract formation on MRI. Pus culture and sensitivity were also studied, and Staphylococcus aureus was found to be the causative organism. Intravenous antibiotics were given. Injection of ceftriaxone 2 gm through an intravenous route every 24 hours was given for 10 days. After antibiotic treatment, surgical debridement was done to clear all the remaining infectious tissue. Sequestrectomy was done to remove dead bony tissue. The wound was kept open for a few days. Later, an artificial bone graft and myocutaneous flap were inserted to close the wound. After all the procedures, the patient was given a combined oral antibiotic regimen of ciprofloxacin and rifampicin for one month. The patient was advised bed rest on discharge and serial follow-up after one month.

## Discussion

Infection of bone is known as osteomyelitis, which predominantly involves the medullary cavity [1]. It can occur in bacterial infections like Staphylococcus aureus, Escherichia coli, Klebsiella, Salmonella, Pseudomonas, Haemophilus influenzae, and group B streptococci. It can also occur due to rare fungal infections. Fungal organisms like Candida and Aspergillus species can cause osteomyelitis by the hematogenous route, through direct spread, and in septic arthritis patients [2]. Fungal osteomyelitis predominantly involves the vertebrae and ribs in adults and the femur and humerus in pediatric patients [3].

Bacterial osteomyelitis can occur through the hematogenous route, traumatic extension, and direct spread from ulcers in diabetic patients. It is usually found in males, predominantly 2-12 years, however, it can be seen in any age group according to specific etiology. Osteomyelitis can be divided into acute bacterial, chronic, fungal, tubercular, and syphilitic osteomyelitis.

Clinical features in acute osteomyelitis include bone pain, swelling, redness, fever, weakness, arthralgia, and tenderness. In chronic osteomyelitis, patients often come with deformity and pus discharge from the sinuses involving the bone. It can be diagnosed with the help of radiological investigations like plain radiographs, ultrasound, CT, and predominantly MRI. On a plain radiograph, osteomyelitis can be seen as a Brodie abscess, which is a lytic lesion, oval, and predominantly in the metaphysis of a long bone such as the tibia. They are surrounded peripherally with sclerotic rims. On MRI, an intra-osseous abscess appears as a thick rim of T1 signal hyper-intensity and hypo-intensity in the central cavitary part, also known as the penumbra sign. This T1 signal peripheral hyper-intensity represents vascularized granulation tissue. Brodie's abscess is usually subacute. It shows peripheral enhancement in contrast imaging [4].

Garre's sclerosing osteomyelitis is found in the mandible, which is chronic. Etiology includes dental caries. It presents as bone formation outside the cortex and inside the periosteum, giving an onion-skin appearance [5]. Acute osteomyelitis on radiograph shows decreased bone density at a particular involved region in the bone known as regional osteopenia. Cortical erosion, trabecular bone loss, and loss of the inner cortex layer, also known as end osteal scalloping, are also present. Newborn formation on the involved bone, also known as a periosteal reaction, can also be seen [6]. There is also evidence of necrotic bone formation known as sequestrum in chronic osteomyelitis. It appears sclerotic with central lucency on a radiograph.

On ultrasound, we can visualize soft tissue involvement. It can also access cellulitis and abscesses in the neighboring soft tissue, sinus tract, and sub-periosteal collection.

On computed tomography, bony details are excellently visualized. Periosteal reaction, a necrotic bone formation that is sequestrum, is visualized on CT as a hyper-dense focus in the medullary cavity. Involucrum represents a dense irregular thickening of the cortex [6,7]. The cloaca is in the form of small gaps in the cortical bone, which help in pus drainage. These findings are usually found in chronic osteomyelitis. There is also evidence of fat stranding, hyper-density in the marrow, and marrow edema.

On MRI, we can visualize all features of soft tissue involvement such as sinus, intraosseous abscess, marrow edema, sequestrum, periosteal reaction, cloaca, and involucrum. It is the most sensitive modality to evaluate soft tissue involvement. The earliest feature of osteomyelitis is bone marrow edema which can be identified earliest on MRI. In the case of active infection, there is a hypointense signal on T1 and a hyperintense signal on T2/T2 fat saturation (Fat-Sat) imaging and proton density fat saturation imaging (PD-Fat-Sat). In the case of fibrosis with an active infection in chronic osteomyelitis, there is a hypointense signal on T1 but heterogeneous intensity on T2/T2 FATSAT and PD Fat-Sat. On MRI, sequestrum appears hypointense on T1, representing dead bone tissue. Cloaca appears as a gap on T2/PD Fat-Sat in the hypointense cortex of bone. The involucrum appears as a hyper-intense signal on T1 [8].

Patients can also go for a bone biopsy to confirm the infection. Bone biopsy can be done with the help of a bone biopsy needle, such as a Jamshidi needle, under the guidance of CT or CT fluoroscopy. Tecnicium-99 bone scintigraphy can be done to detect any small foci of disease. Indium-111-labeled WBC scintigraphy can be done in cases of osteomyelitis due to diabetes and ulcers of chronically bedridden patients [9,10]. Gallium-67 scintigraphy can be done to identify the inflammatory focus and inactive infection. However, it does not give proper details of bone and cannot differentiate associated soft tissue infection. Positron emission tomography-computed tomography (PET-CT) with fluorodeoxyglucose (FDG) uptake is better than scintigraphy in detecting and confirming chronic osteomyelitis.

Treatment

In cases of acute osteomyelitis, intravenous antibiotics should be given after performing a culture for the sensitivity of a particular disease-causing organism and should be continued for six weeks. Bedrest, immobilization of the involved limb, and analgesics should also be given for 15 days. In cases of chronic infection, drainage of abscess and surgical debridement should be done. Sequestrectomy should be done. In cases of extreme infections, amputation of the limb can also be done so that the infection cannot involve other parts of the body.

## Conclusions

This case report highlights the importance of bodily infections, which can progress to the bones and cause osteomyelitis in children. So the timely detection of infections and appropriate management can prevent osteomyelitis. This is a common infection but if it becomes chronic, it can lead to weakened bones, ultimately creating deformity and hampering a child's growth. It also leads to overall physical, social, and developmental, stigma to the children. So, the help of advanced imaging and early diagnosis with modalities like MRI can decrease the burden of such diseases in society. Early management can also hamper the progress of such infections and provide a better quality of life index to children.
